# Acute colonic variceal bleeding treated with coil-assisted retrograde transvenous obliteration following atezolizumab–bevacizumab therapy for advanced hepatocellular carcinoma: A case report

**DOI:** 10.1097/MD.0000000000051084

**Published:** 2026-10-16

**Authors:** Sung Eun Park, Hyeon Uk Kwon, Sa Hong Jo, Sung Gong Lim, Ji Yoon Kwak, Ji Hee Han, Hankyu Jeon, Ra Ri Cha, Jae Min Lee, Hyun Jin Kim, Sang Soo Lee

**Affiliations:** a Department of Radiology, Gyeongsang National University School of Medicine and Gyeongsang National University Changwon Hospital, Changwon, Republic of Korea; b Institute of Medical Sciences, Gyeongsang National University, Jinju, Republic of Korea; c Department of Internal Medicine, Gyeongsang National University School of Medicine and Gyeongsang National University Changwon Hospital, Changwon, Republic of Korea; d Department of Internal Medicine, Gyeongsang National University School of Medicine and Gyeongsang National University Hospital, Jinju, Republic of Korea.

**Keywords:** atezolizumab, bevacizumab, case report, coil-assisted retrograde transvenous obliteration, colonic variceal bleeding, hepatocellular carcinoma

## Abstract

**Rationale:**

Colonic variceal bleeding is a rare but potentially life-threatening manifestation of portal hypertension. Bevacizumab therapy is associated with an increased risk of variceal bleeding, but bleeding from ectopic varices is less well recognized.

**Patient concerns:**

A 66-year-old man with alcohol-related cirrhosis and advanced hepatocellular carcinoma developed hematochezia on the day following the initiation of atezolizumab plus bevacizumab therapy.

**Diagnoses:**

Acute ascending colonic variceal bleeding in the setting of portal hypertension and portal vein thrombosis.

**Interventions:**

Coil-assisted retrograde transvenous obliteration (CARTO) was performed via the right common femoral vein. The portocolonic shunt was embolized using detachable coils and gelfoam slurry.

**Outcomes:**

Hematochezia ceased immediately after the procedure, and the patient remained hemodynamically stable without further transfusion. Follow-up computed tomography confirmed successful embolization of the variceal complex without residual shunt.

**Lessons:**

CARTO may be an effective treatment option for colonic variceal bleeding, particularly in patients with portal vein thrombosis or complex portosystemic shunts. Clinicians should remain aware of the potential for bleeding from ectopic varices in patients receiving bevacizumab-based therapy.

## 1. Introduction

Ectopic varices are portosystemic collaterals located outside the gastroesophageal region and account for up to 5% of all variceal bleeding.^[1-4]^ Among ectopic varices, colonic varices are particularly rare and constitute only a small proportion.^[3]^ Given their low prevalence, no standardized treatment strategies have been established, and management is typically individualized.^[5]^ Endoscopic, surgical, and radiologic approaches have been described; however, outcomes vary according to the underlying vascular anatomy and hemodynamic factors.^[6,7]^

Coil-assisted retrograde transvenous obliteration (CARTO) is a modified retrograde transvenous obliteration (RTO) technique used for the treatment of gastric and other ectopic varices.^[2,8]^ However, its application in colonic variceal bleeding has been reported only rarely.^[2,9]^

In patients with cirrhosis, increased intrahepatic vascular resistance and increased splanchnic blood flow lead to portal hypertension and the development of portosystemic collateral vessels. The anatomy and hemodynamics of these collateral pathways are associated with hepatic functional reserve, clinical manifestations, and prognosis. Colonic and other ectopic varices may develop when mesenteric venous flow is diverted through atypical portosystemic pathways. Portal vein thrombosis may further alter portal hemodynamics and promote collateral formation. In patients with hepatocellular carcinoma (HCC), concomitant portal vein thrombosis, whether bland or tumor-related, may further alter portal hemodynamics, complicate the management of variceal bleeding, and adversely affect clinical outcomes. However, outcome data specific to patients with colonic varices remain limited.^[2,10]^

Bevacizumab inhibits vascular endothelial growth factor-mediated angiogenesis and may compromise endothelial integrity and vascular repair, thereby increasing susceptibility to hemorrhagic complications. This risk is particularly relevant in patients with cirrhosis and portal hypertension who have preexisting varices. In the IMbrave150 trial, patients were required to undergo endoscopic evaluation for varices within 6 months before treatment, and those with untreated or incompletely treated esophagogastric varices with bleeding or a high risk of bleeding were excluded.^[11]^ However, routine esophagogastroduodenoscopy does not evaluate most ectopic varices, and the risk of ectopic variceal bleeding during bevacizumab-containing therapy remains poorly defined.^[12]^ Only a limited number of cases of ectopic variceal rupture during atezolizumab–bevacizumab therapy have been reported.^[2,13]^

We report a case of acute ascending colonic variceal bleeding that developed shortly after initiation of atezolizumab plus bevacizumab therapy in a patient with advanced HCC and portal vein thrombosis, successfully treated with coil-assisted retrograde transvenous obliteration.

## 2. Case presentation

A 66-year-old man with alcohol-related cirrhosis (Child–Pugh class A) and advanced HCC was admitted for systemic therapy. He had previously undergone 5 sessions of transarterial chemoembolization and received palliative radiotherapy. Systemic therapy with atezolizumab plus bevacizumab was subsequently initiated.

One day after the first administration of atezolizumab plus bevacizumab, the patient developed hematochezia, and his hemoglobin level decreased from 9.3 to 8.0 g/dL. Initial esophagogastroduodenoscopy and sigmoidoscopy did not reveal the source of bleeding. Because hematochezia recurred, repeat endoscopic examinations were performed; however, the bleeding source could not be identified. Despite repeated packed red blood cell transfusions, his hemoglobin level further decreased to 6.5 g/dL. He subsequently developed hemodynamic instability, with a blood pressure of 75/40 mm Hg and tachycardia. At the time of hemodynamic deterioration, laboratory tests revealed marked leukocytosis (33,880/µL) and thrombocytopenia (70 × 10^3^/µL). The marked leukocytosis was considered most likely to represent reactive stress leukocytosis associated with acute blood loss and hemodynamic instability, as there were no clinical signs of infection.

Contrast-enhanced computed tomography (CT) demonstrated liver cirrhosis with portal vein thrombosis, a gastrorenal shunt, and an engorged ileocolic vein adjacent to the ascending colon, suggestive of a portocolonic shunt (Fig. 1). Repeat colonoscopy revealed a suspected variceal lesion in the ascending colon (Fig. 2A). Endoscopic clipping was attempted; however, hemostasis was not achieved (Fig. 2B). Given the persistent bleeding in the setting of a portocolonic shunt, CARTO was performed.

**Figure 1.**
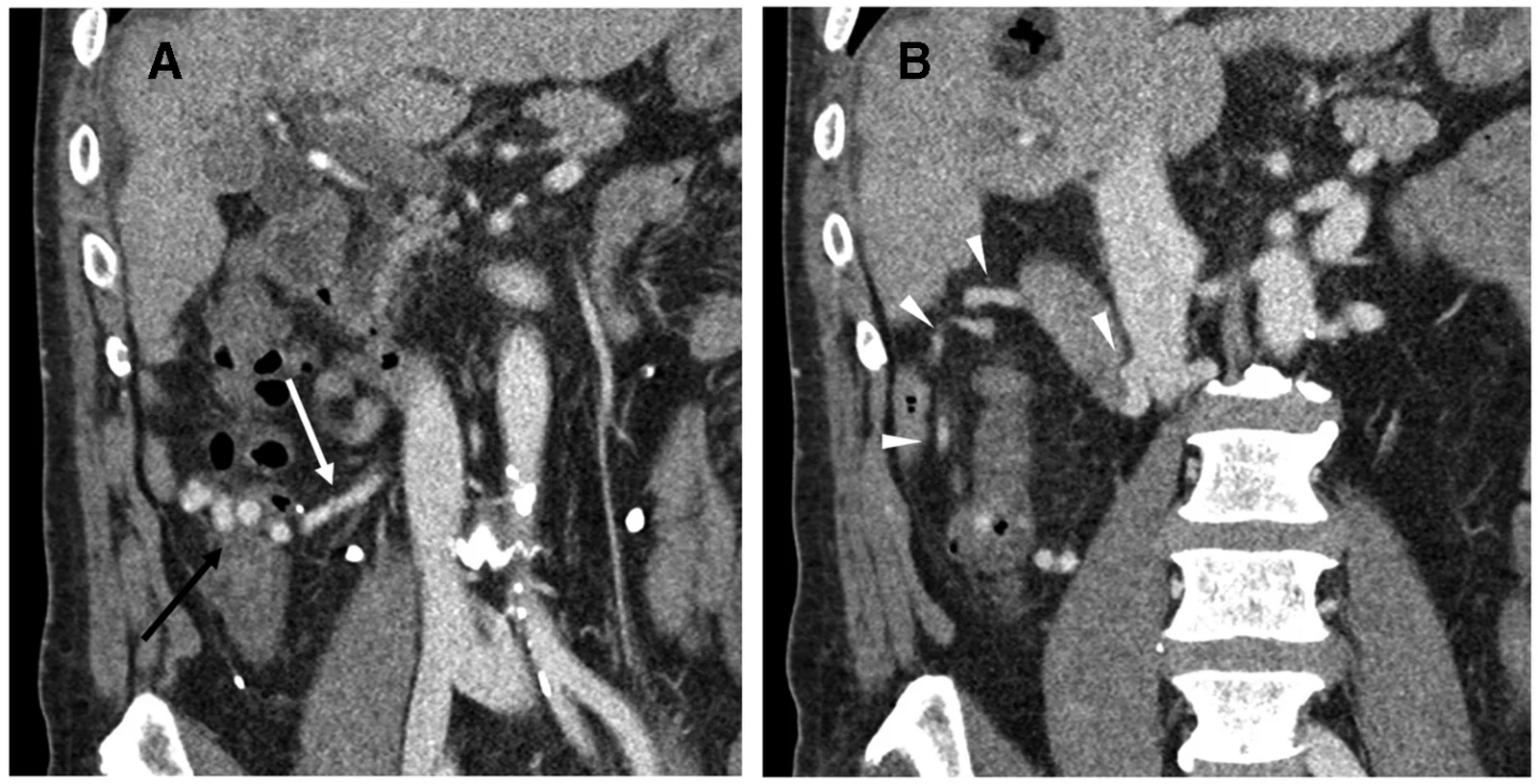
Preprocedural contrast-enhanced computed tomography findings of the portocolonic variceal complex. (A) Coronal portal venous phase image showing a colonic varix (black arrow) and an engorged ileocolic vein adjacent to the ascending colon (white arrow). (B) Coronal image demonstrating a portosystemic collateral pathway connecting the ileocolic vein to the inferior vena cava (arrowheads).

**Figure 2.**
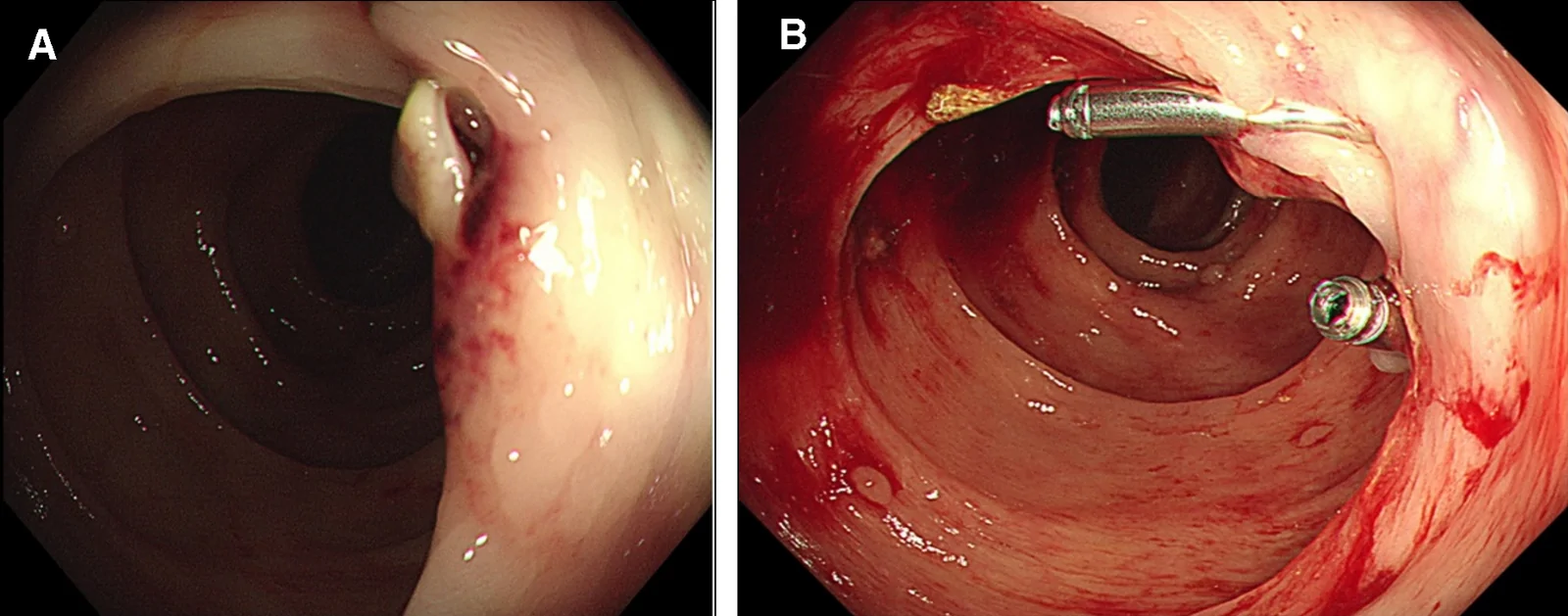
Colonoscopic findings of ascending colonic variceal bleeding. (A) Colonoscopic image showing a submucosal variceal lesion with a white nipple sign in the ascending colon, indicating recent variceal hemorrhage. (B) Endoscopic clipping was attempted; however, active bleeding persisted despite clip placement.

Under ultrasound guidance, the right common femoral vein was punctured, and a 6-Fr sheath (Flexor Ansel guiding sheath; Cook, Bloomington) was inserted. Venography demonstrated a portocolonic shunt originating from the inferior vena cava (Fig. 3A). The shunt was selectively catheterized using a 5-Fr Cobra catheter (Merit Medical, South Jordan), and the efferent shunt was accessed using a double microcatheter system consisting of a Progreat Lambda 1.9-Fr (Terumo, Tokyo, Japan) and a Progreat Alpha 2.0-Fr (Terumo, Tokyo, Japan) with 0.016-inch microwires. The microcatheters were positioned in the distal and proximal portions of the shunt. Embolization of the proximal shunt was achieved using 5 detachable coils (two Concerto; Medtronic, Irvine, and 3 Interlock; Boston Scientific, Maple Grove; Fig. 3B). The colonic varix was subsequently embolized using gelfoam slurry through the distal microcatheter. Contrast stasis within the varix was confirmed, and cone-beam CT demonstrated complete filling of the variceal complex (Fig. 3C). The procedure was completed without immediate complications.

**Figure 3.**
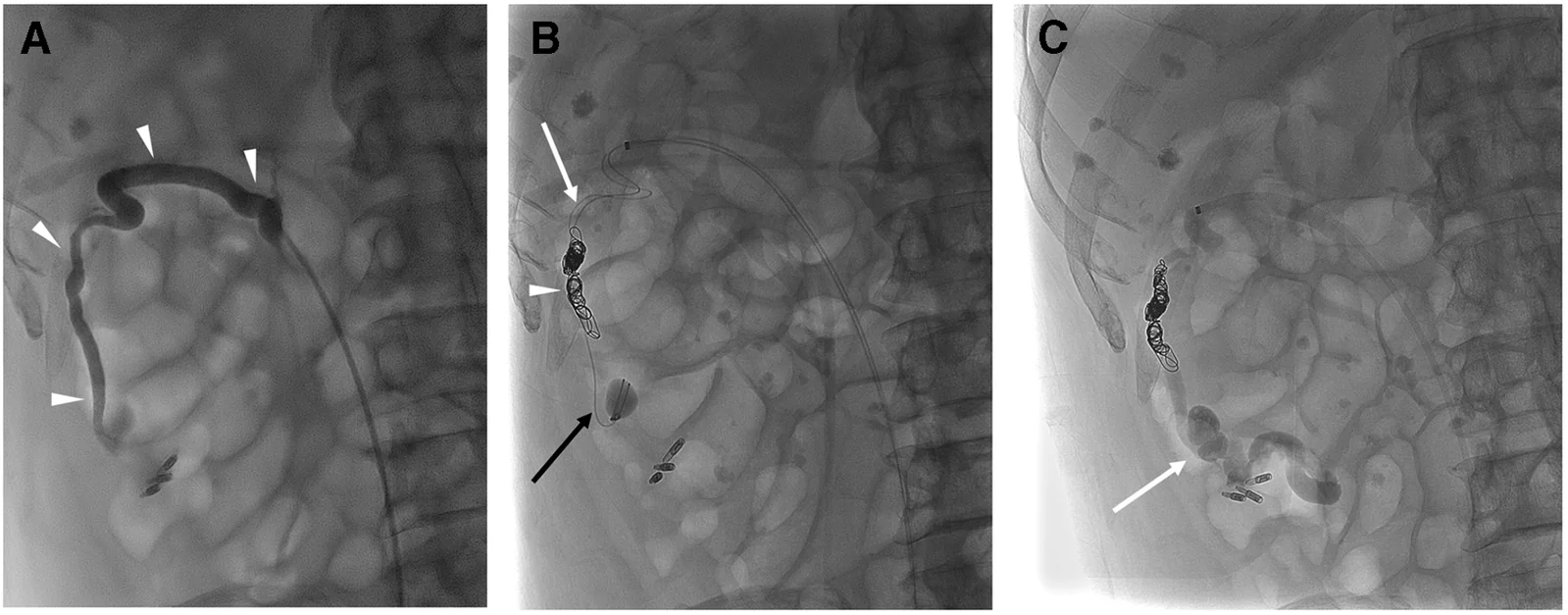
Coil-assisted retrograde transvenous obliteration of the ascending colonic varix. (A) Venography demonstrating the portocolonic shunt draining into the inferior vena cava (arrowheads). (B) A double-microcatheter technique was used. The proximal microcatheter (white arrow) was used for coil deployment, and the distal microcatheter (black arrow) was used for gelfoam injection. Multiple detachable coils (arrowhead) were deployed to occlude the efferent shunt. (C) Postembolization venography showing contrast stasis within the variceal complex (arrow), confirming successful obliteration.

After CARTO, hematochezia ceased, and the patient remained hemodynamically stable without further transfusion. Follow-up contrast-enhanced CT demonstrated embolization of the mesenteric venous complex adjacent to the ascending colon, consistent with successful obliteration of the portocolonic varix and without evidence of a residual shunt (Fig. 4).

**Figure 4.**
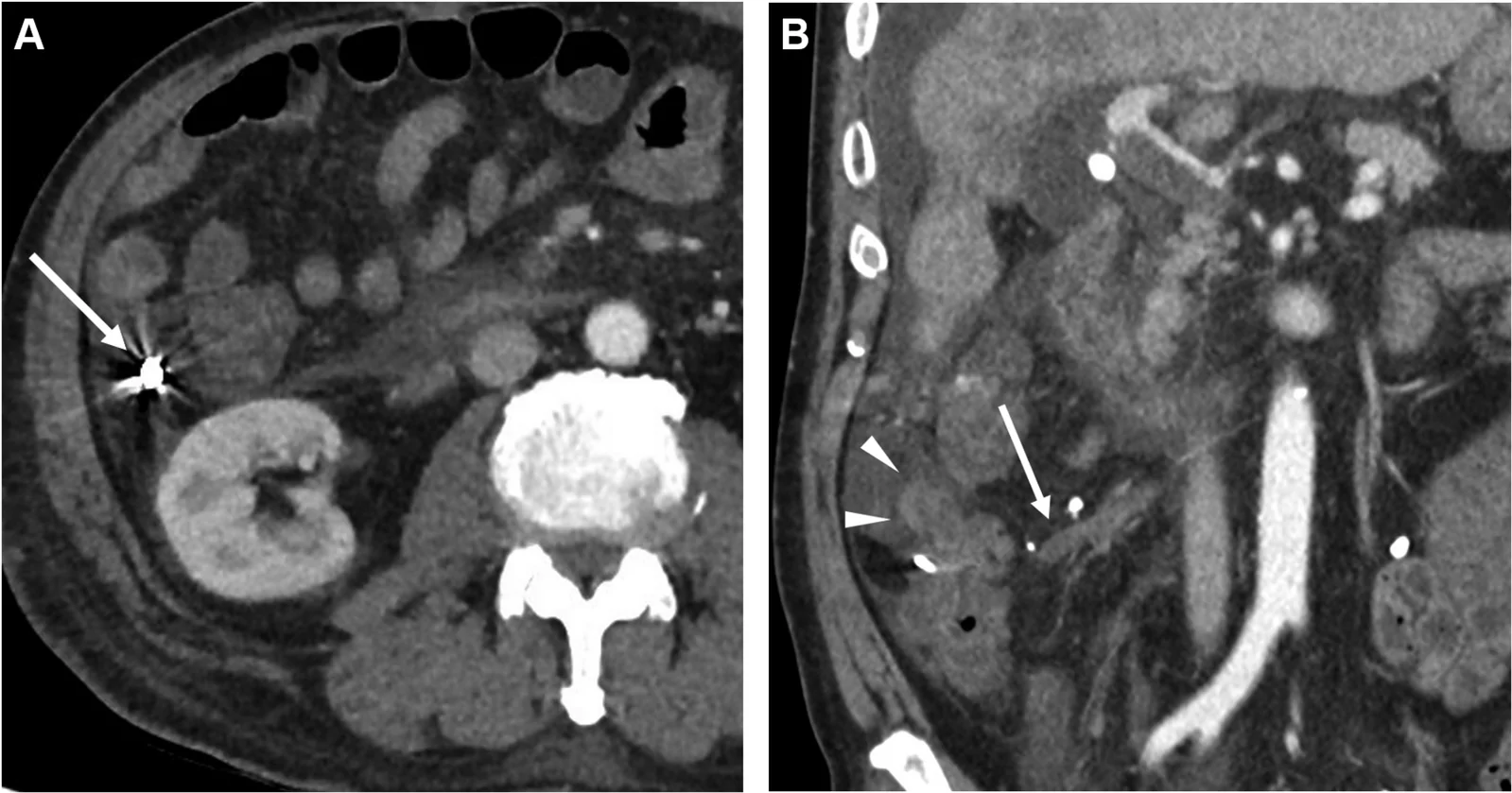
Follow-up contrast-enhanced computed tomography after coil-assisted retrograde transvenous obliteration. (A) Axial image demonstrating coil embolization of the portocolonic shunt adjacent to the ascending colon (arrow). (B) Coronal image showing complete obliteration of the colonic varix (arrowheads) and thrombosis of the engorged ileocolic vein (arrow).

## 3. Discussion

Colonic varices are rare and constitute a small proportion of ectopic varices. Once bleeding occurs, it can be life-threatening and challenging to manage.^[1,7]^ Given their low prevalence, no standardized treatment strategies have been established.^[2,5]^ Endoscopic therapy is often attempted as an initial approach but may fail due to poor visualization, hemodynamic instability, or complex vascular anatomy.^[6]^ Surgical resection is associated with high morbidity in patients with cirrhosis, and transjugular intrahepatic portosystemic shunt placement may not adequately control bleeding when local venous obstruction is present.^[2]^

CARTO offers a minimally invasive alternative by obliterating the variceal outflow tract and thereby reducing variceal pressure. Although this technique has been increasingly applied in the management of gastric and other ectopic varices, only a limited number of cases of colonic variceal bleeding treated with CARTO have been reported.^[8,9]^

The technical success of RTO procedures depends on the ability to selectively access and occlude the efferent shunt. In the present case, CARTO was chosen because the portocolonic shunt was tortuous and associated with portal vein thrombosis. Although balloon-occluded RTO is the most established technique, it requires advancement of a relatively large balloon catheter into the efferent vein, which may be technically challenging in small or tortuous vessels.^[14]^ CARTO utilizes a microcatheter system, often employing a double microcatheter technique, which provides improved flexibility and trackability in complex vascular anatomy.^[15]^ Compared with plug-assisted RTO, which is limited by plug size and requires a large-bore sheath, CARTO allows more adaptable coil deployment across various shunt anatomies.^[16]^ This approach enables precise embolization at the distal or narrow portion of the shunt, thereby minimizing the risk of embolic migration and facilitating effective obliteration of the variceal complex.^[8]^

Colonic varices are rare forms of ectopic varices and represent only a small proportion of portosystemic collaterals in patients with portal hypertension.^[16]^ When bleeding occurs, it can result in massive and potentially life-threatening hematochezia. Unlike gastric varices, ectopic varices frequently involve complex and unpredictable collateral pathways, such as the mesenterogonadal or portocolonic shunts observed in this case.^[16]^ Endoscopic management, including clipping or band ligation, can be technically challenging and carries a risk of rebleeding because of the deep location and high-flow characteristics of these thin-walled vessels. In this context, CARTO may serve as a valuable endovascular option, particularly when endoscopic treatment is unsuccessful or technically difficult.

Another notable aspect of this case is that the bleeding occurred shortly after initiation of atezolizumab plus bevacizumab therapy. Bevacizumab-based treatment is known to increase the risk of variceal bleeding, and screening for gastroesophageal varices is recommended before therapy.^[11]^ However, the risk of bleeding from ectopic varices is less well recognized in clinical practice.^[13]^ This case highlights that clinicians should be aware of the potential for bleeding from ectopic varices, not only gastroesophageal varices, particularly in patients receiving bevacizumab-based therapy.

This case report has limitations. As it describes a single patient, the findings cannot be generalized. Furthermore, although colonic variceal bleeding occurred shortly after the initiation of atezolizumab plus bevacizumab therapy, the temporal association alone does not establish causality.

## 4. Conclusions

CARTO may represent a feasible and effective treatment option for colonic variceal bleeding, particularly in patients with complex portosystemic shunts or portal vein thrombosis. Clinicians should be aware of the potential for bleeding from ectopic varices in patients receiving bevacizumab-based therapy. Further prospective studies are needed to confirm the safety and efficacy of CARTO for colonic variceal bleeding.

## Author contributions

**Data curation:** Ji Yoon Kwak, Ji Hee Han, Hankyu Jeon, Ra Ri Cha, Jae Min Lee, Hyun Jin Kim.

**Investigation:** Sung Eun Park, Sa Hong Jo, Sung Gong Lim, Ji Yoon Kwak, Ji Hee Han, Hankyu Jeon, Ra Ri Cha, Jae Min Lee, Hyun Jin Kim.

**Methodology:** Sung Eun Park, Sa Hong Jo, Sung Gong Lim.

**Writing – original draft:** Sung Eun Park, Hyeon Uk Kwon, Sang Soo Lee.

**Writing – review & editing:** Sung Eun Park, Hyeon Uk Kwon, Sa Hong Jo, Sung Gong Lim, Ji Yoon Kwak, Ji Hee Han, Hankyu Jeon, Ra Ri Cha, Jae Min Lee, Hyun Jin Kim, Sang Soo Lee.
